# Development of a Digital Twin for the Prediction and Control of Supersaturation during Batch Cooling Crystallization

**DOI:** 10.1021/acs.iecr.3c00371

**Published:** 2023-07-03

**Authors:** Ryan Leeming, Tariq Mahmud, Kevin J. Roberts, Neil George, Jennifer Webb, Elena Simone, Cameron J. Brown

**Affiliations:** † School of Chemical and Process Engineering, University of Leeds, Leeds LS2 9JT, U.K.; ‡ Syngenta, Jealott’s Hill, Bracknell RG42 6EY, U.K.; § Department of Applied Science and Technology, Politecnico di Torino, Torino 10129, Italy; ∥ CMAC Future Manufacturing Research Hub, University of Strathclyde, Glasgow G1 1RD, U.K.

## Abstract

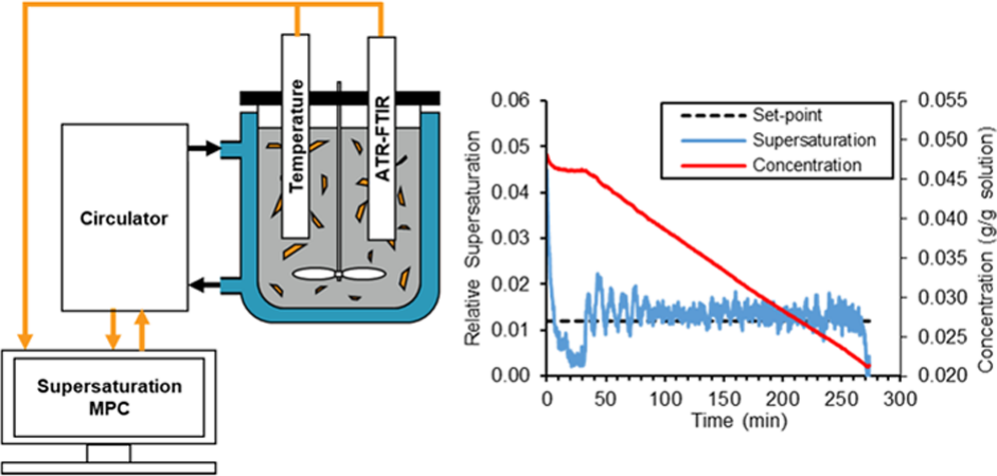

Fine chemicals produced via batch crystallization with properties dependent on the crystal size distribution require precise control of supersaturation, which drives the evolution of crystal size over time. Model predictive control (MPC) of supersaturation using a mechanistic model to represent the behavior of a crystallization process requires less experimental time and resources compared with fully empirical model-based control methods. Experimental characterization of the hexamine–ethanol crystallization system was performed in order to collect the parameters required to build a one-dimensional (1D) population balance model (PBM) in gPROMS FormulatedProducts software (Siemens-PSE Ltd.). Analysis of the metastable zone width (MSZW) and a series of seeded batch cooling crystallizations informed the suitable process conditions selected for supersaturation control experiments. The gPROMS model was integrated with the control software PharmaMV (Perceptive Engineering Ltd.) to create a digital twin of the crystallizer. Simulated batch crystallizations were used to train two statistical MPC blocks, allowing for in silico supersaturation control simulations to develop an effective control strategy. In the supersaturation set-point range of 0.012–0.036, the digital twin displayed excellent performance that would require minimal controller tuning to steady out any instabilities. The MPC strategy was implemented on a physical 500 mL crystallizer, with the simulated solution concentration replaced by in situ measurements from calibrated attenuated total reflection–Fourier transform infrared (ATR-FTIR) spectroscopy. Physical supersaturation control performance was slightly more unstable than the in silico tests, which is consistent with expected disturbances to the heat transfer, which were not specifically modeled in simulations. Overall, the level of supersaturation control in a real crystallizer was found to be accurate and precise enough to consider future adaptations to the MPC strategy for more advanced control objectives, such as the crystal size.

## 1 Introduction

### 1.1 Background

A variety of fine chemical products including pharmaceuticals and agrochemicals are produced through batch cooling crystallization, optimizing the process conditions to reach a high yield and purity of product crystals. It is also important to achieve good particle physical properties, such as a specific crystal size distribution (CSD), in order to optimize product performance properties that are connected to size and shape, such as the dissolution rate. Downstream operations including filtration and drying are also impacted by the product CSD and can be hindered significantly by the overabundance of fine material.

In the case of batch cooling crystallization, the objective for control is usually to find an optimal temperature profile that will lead to the desired CSD. The supersaturation generated during cooling is directly connected to the key rate mechanisms of crystal nucleation and growth, which drive the crystallization process. The creation of an accurate supersaturation control system has been robust for over a decade since advancements in analytical technology have allowed this property to be monitored and controlled directly.^1,2^

Seed crystals are typically introduced to tailor a crystallization process to achieve specific CSD properties; however, they also bring potential challenges to the control of supersaturation. For example, a high seed mass can inhibit nucleation, leading to a completely different shape of the product CSD.^3^ The supersaturation trajectory can also be altered by the quality of seed crystals, due to the effects on crystal growth caused by uneven surfaces formed during milling.^4^ Surface effects such as these are usually repaired through the preparation of seed slurries for the feed, which treat these problems through Ostwald ripening.^5^ Seed loading has also been observed as a key factor during supersaturation control, which can affect the frequency of nucleation events.^6^

Measurement-based control schemes have been applied using techniques such as attenuated total reflection (ATR)–Fourier transform infrared (FTIR) spectroscopy, which can provide an accurate measurement of solution concentration and therefore supersaturation. In addition to alternatives for concentration measurement including Raman and ATR-UV/Vis spectroscopy, there exist a range of well-established techniques for in situ particle imaging and size analysis, including focused-beam reflectance measurement (FBRM) and particle vision and measurement (PVM).^7,8^

In situ concentration measurement can then be used to either maintain a constant level of supersaturation or follow a predetermined profile. These controllers operate using simple algorithms to manipulate the temperature of the heating/cooling jacket around a crystallization vessel using a feedback loop.^9−11^ For example, Fujiwara et al.^12^ calibrated a proportional-integral (PI) temperature controller to follow a set supersaturation profile close to the metastable limit, therefore ensuring optimum crystal growth. This approach to control is referred to as direct design, which attempts to overcome uncertainty on the kinetic parameters of a crystallization system using an engineering understanding of the metastable zone.^13^

A major downside to this approach is the empirical nature of the controller development, requiring large amounts of experimental data to quantify mechanisms that in practice vary widely depending on the chosen operating conditions. Khan et al.^2^ studied supersaturation control during the batch cooling crystallization of l-glutamic acid at both 20 and 250 L scales. Each scale required a different cooling profile to maintain the same level of supersaturation, and variation in the product CSD was influenced by the more prevalent secondary nucleation occurring in the smaller scale.

The progression from measurement-based to model-based control has helped to quantify the necessary understanding of a batch crystallization process in a more generally applicable form. Ideally, models would be solely derived from physical concepts, but in real cases, there is always a partial empirical contribution. So-called “first-principles” or mechanistic models use experimentation to aid parameter estimation, an improvement over fully empirical “batch-to-batch” models that require extensive experimental characterization to function sufficiently.^14−16^ The mechanistic approach to crystallization modeling is focused around connecting material and energy balances to a crystal population balance model (PBM) in order to optimize a function like the supersaturation profile.^17^

The PBM describes the number and size of particles through the number density function *n*(*L*), including terms for nucleation, growth, breakage, and agglomeration that can be included or removed as required. A one-dimensional (1D) PBM can be written in the following simplified form for a batch crystallizer with no inward or outward flow and a growth rate *G* that is independent of crystal size
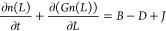
1The number density can also be expressed as a volume density function *v*(*L*) using a specific crystal’s characteristic length and shape factor^18^
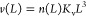
2Empirical models benefit from the use of more easily gathered off-line measurements, such as in the batch-to-batch iterative model developed by Forgione et al.^16^ Improved supersaturation control was achieved by updating the cooling profile after comparison with previous batch data. However, as a feedforward mode of control, it was susceptible to being impacted negatively by external disturbances to temperature dynamics, which can generate significant model mismatch. Griffin et al.^19^ paired ATR-FTIR with chord length and particle count measurements from FBRM to develop a dynamic model for controlling the average crystal size and yield within set batch times. This was made achievable by limiting the dimensionality of the model, compromising between computational efficiency and the level of detail and accuracy that a PBM could capture.

A stronger form of feedforward control that allows a PBM to be implemented is model predictive control (MPC), which involves the generation of control moves made to the manipulated variable that most efficiently reach and maintain a controlled variable set-point.^10^ MPC benefits from being able to handle complex, dynamic multiple input/output systems, making it superior over other forms of control for batch cooling crystallization due to the change in nucleation and growth rates as the supersaturation varies.^20^ That being said, most successful MPC applications for controlling crystal size have been shown for continuous processes rather than batch.^21−23^ An effective real-time process monitoring and control approach, built using the software PharmaMV (Perceptive Engineering Ltd.), was demonstrated by Tahir et al.^23^ to control the median crystal size within a continuous oscillatory flow crystallizer. This technique benefitted from the steady-state nature of continuous processes where a constant set-point for the median size could be applied. However, this would need to be expanded upon for batch crystallization, given that the crystal size is expected to change constantly as it develops toward a certain end-point throughout the process.

Most industries are undergoing a digital transformation, using technology in new ways to improve efficiency. For the fine chemical industry, there exists the concept of Pharma 4.0, built on a foundation of digital maturity to develop resources, organization, processes, information systems, and the working culture.^24^ Regarding crystallization, the use of technology has been studied thoroughly to provide more accurate process models. Szilágyi and Nagy^25^ showed how parallel GPUs running a high-resolution finite volume technique improved calculation speed significantly, enough for real-time resolution of a multidimensional PBM. Camacho Corzo et al.^26^ used computational fluid dynamics (CFD) to investigate the hydrodynamics inside a batch crystallizer, suggesting that these simulations could be paired with a morphological PBM to create a more robust representation of the process.^27^

Another recent advancement for MPC of crystallization processes is the use of open-loop simulations with the PBM to train machine-learning control algorithms, such as in the study by Zheng et al.^28^ They demonstrated how a recurrent neural network (RNN) model trained using a semiempirical PBM could improve the computational efficiency of MPC to achieve an optimal target product yield and crystal size. However, advanced control methods such as this have only been tested in silico, noting particular difficulties that may arise when applying these MPC methods on a real crystallizer, such as the accuracy of available PAT. A reinforced-learning approach to improve the control of temperature, supersaturation, and crystal size trajectories was taken by Benyahia et al.^29^ for the batch cooling crystallization of paracetamol in water. This control method showed a significant improvement over MPC for controlling the crystal size; however, MPC performed similarly for both temperature and supersaturation, therefore remaining a relevant and applicable approach to crystallization process control.

Continuing the Pharma 4.0 vision, this paper presents a methodology for the development of an MPC strategy involving the formation of a digital twin^30^ using two commercial pieces of software. The mechanistic process modeling tool gPROMS FormulatedProducts (Siemens-PSE Ltd.) has been coupled with the in-line monitoring and control tool PharmaMV (Perceptive Engineering Ltd.) to provide a way of simulating batch cooling crystallization processes under the control of both supersaturation and the product CSD. After assessing the in silico performance of the MPC strategy using simulated data, the software platform was connected to a physical lab-scale crystallization setup to control real batch operations for validation.

The proposed methodology would reduce the experimental time needed to create a robust model for industrial crystallization systems, particularly during new product development. The digital twin can be utilized to train the predictive controllers in PharmaMV without expending any physical resources, efficiently capturing the behavior of a given crystallization system. Communication between the software and a variety of providers of crystallization equipment and PAT is already established, allowing a developed MPC strategy to be physically implemented on industrial equipment setups with minimal effort.

### 1.2 Approach to Control Strategy Development

The general approach adopted toward the development of the control strategy reported in this paper can be summarized into four main stages. This approach was taken after the selection of a model solute–solvent system, which would reduce the complexity of the initial process model development. The selection criteria included both being readily available and having a good temperature dependence of solubility to ensure a high yield from large-scale crystallization. Most importantly, the system was to have a crystal morphology resulting in equant growth in all directions, making it suitable for a 1D PBM.

Stage 1 involved performing lab-scale experiments to understand the key crystallization mechanisms for the selected system, including the characterization of the metastable zone width (MSZW) and seeded batch cooling crystallizations. The process data gathered during this step was used for the construction of the process model and later for validation purposes.

Stage 2 involved the parameterization of a 1D PBM in gPROMS FormulatedProducts software, using solubility and nucleation/growth kinetic data from the literature.^31^ This model was used to simulate batch crystallizations in a vessel with comparable dimensions to experiments, predicting the final CSDs produced when following set cooling profiles. Comparisons between the predicted and experimental product CSDs and supersaturation profiles were used for model validation, and the growth rate kinetics obtained from the literature were reestimated using measured concentration data.

Stage 3 coupled the gPROMS process model with the control software PharmaMV to formulate the MPC strategy for supersaturation control. Two MPC controllers which connected supersaturation with the cooling profile during a batch were trained using the process model, allowing in silico simulations of supersaturation control at varying levels to be performed.

Stage 4 involved the validation of these simulations, where the MPC strategy in PharmaMV was connected to a physical batch crystallizer with appropriate in situ measurement techniques to replace those simulated by gPROMS. The MPC strategy’s performance between in silico and physical supersaturation control was compared to make a final assessment of the current strategy’s capabilities.

## 2 Materials and Methodology

### 2.1 Selection of Solute–Solvent System

The material chosen for this study was hexamethylene tetramine (hexamine), widely used in applications including the treatment of urinary tract infections and the manufacture of fuel tablets that burn smokelessly.^32^ Hexamine was suitable for study due to its single growth form {110}, developing a rhombic dodecahedron morphology as determined through the Bravais–Friedel–Donner–Harker (BFDH) method.^33^ This is due to its highly symmetrical, body-centered cubic unit cell resulting in relatively equant growth in all directions, therefore being well represented by a 1D PBM (i.e., well represented by a sphere).

Hexamine’s solubility in pure water increases as temperature decreases, making it an unsuitable solvent for batch cooling crystallization. For this reason, pure ethanol was chosen instead to fit the more conventional solubility relationship, at the cost of a slightly reduced potential yield.^34^ Myerson et al.^31^ reported power law rate expressions for nucleation and growth of hexamine in various solvents; the data for crystallization from ethanol is summarized in Table 1.

**Table 1:** Solubility, Nucleation Rate, and Growth Rate Expressions for Hexamine–Ethanol Crystallization System Reported by Myerson et al.^31^

solubility (g (g solution)^−1^)	C* = 1.373 × 10^–2^ + 5.729 × 10^–4^*T* + 6.707 × 10^–6^*T*^2^
nucleation rate (#/s·m^3^ solvent)	*J* = (5.0 ± 2.0) × 10^13^Δ*C*^2.6 ± 0.9^
growth rate (m s^–1^)	*G* = 2.6 × 10^–2^Δ*C*^1.95^

Although unspecified in their study, it was assumed that the nucleation expression would most closely represent secondary nucleation, given its determination via continuous crystallization. Additionally, the lack of terms that account for agitation and slurry density suggested a poorly accurate description of the system’s behavior. However, this was a reasonable starting point and posed an important challenge to the MPC strategy’s performance. If a good level of control could be maintained despite slight differences between the simulated and real crystallization behavior, it would be a useful tool for industrial applications that typically face unpredictable disturbances.

In contrast to the widely recognized mechanism of agglomeration through particle collisions, hexamine exhibits an interesting agglomeration mechanism involving nucleation, typically referred to as “primary” agglomeration.^35−38^ Dendritic growth following from nucleation on the corners of hexamine’s morphology leads to clusters of around 6–7 crystals, which incorporate solvent inclusions into the structure upon further crystal growth. Although this mechanism has been thoroughly understood through the molecular modeling work by Nguyen et al.,^39^ the agglomeration rate has not yet been quantified sufficiently for application in a PBM. Therefore, this poses an interesting challenge to the performance of the MPC strategy when agglomeration is not modeled.

### 2.2 Experimental Setup for MSZW Characterization and Seeded Batch Crystallization

The crystallization system consisted of hexamine (purity > 99%, Scientific Laboratory Supplies) recrystallized from solution in pure ethanol (purity > 99.8%, Sigma-Aldrich).

**Figure 1:**
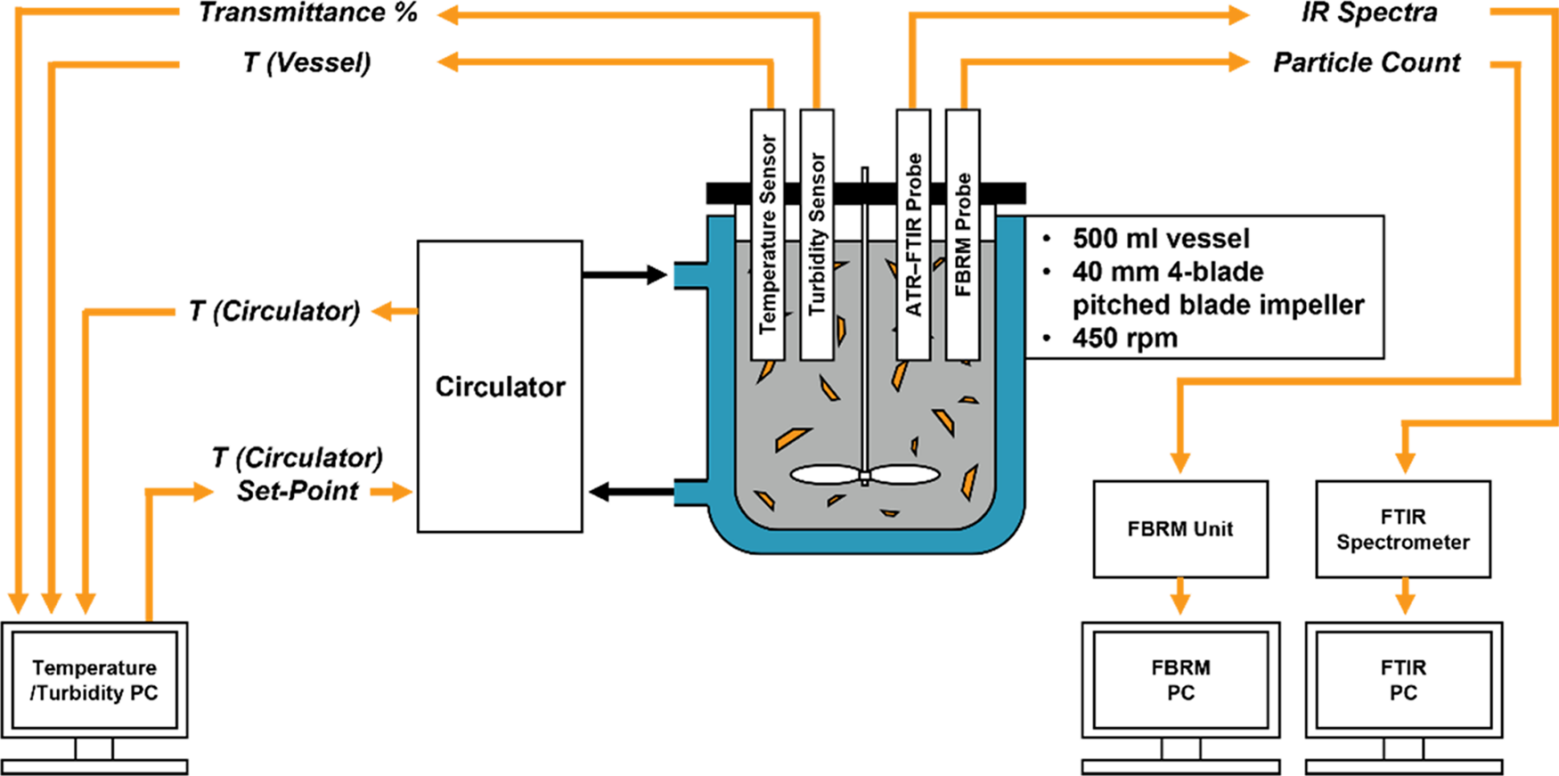
Schematic of the 500 mL jacketed crystallizer and associated in situ measurement technology.

Figure 1 shows a schematic of the experimental setup used to measure the MSZW and carry out seeded batch crystallizations. A Radleys 500 mL unbaffled jacketed glass crystallization vessel with a dish-shaped bottom was clamped to a supporting frame alongside a Radleys RS50 Control Overhead Stirrer, fitted with a 40 mm diameter 4-blade pitched blade impeller. An agitation speed of 450 rpm was used in all batch experiments. The temperature within the vessel was controlled using a Huber Ministat 230 thermostatic bath (operated via in-house LabVIEW software) to circulate water through the jacket and monitored with a PTFE Pt-100 temperature probe connected to the Huber Ministat. An in-house built turbidimetric fiber-optic probe^9^ was inserted into the vessel to measure the solution turbidity and determine the clear/cloud points during heating/cooling. A Mettler Toledo Lasentec S400A FBRM probe was used to measure particle count, while an ATR-FTIR probe connected to an ABB MB3000 FTIR spectrometer was used to measure solution concentration. The method for calibration of the IR spectra to concentration is detailed in the following section.

### 2.3 ATR-FTIR Calibration Model Using HorizonMB

In order to determine the concentration in situ during crystallization experiments, the IR spectra of hexamine–ethanol solutions at varying temperatures and concentrations were measured using an ABB MB3000 FTIR spectrometer. A partial least squares (PLS) regression model was calibrated against the known solution concentrations within the accompanying software, HorizonMB.

For each concentration selected, the appropriate amount of hexamine was dissolved in 400 g of ethanol and heated to approximately 5 °C above the saturation temperature. Once the hexamine had been fully dissolved, spectra were measured every minute, performing 16 scans in the range of 700–1600 cm^–1^ at a resolution of 4 cm^–1^. After holding the temperature for 10 min, the solution was rapidly cooled in steps of 2 °C, holding at each temperature for 10 min. This procedure continued until primary nucleation occurred, and any spectra measured at a constant temperature before the point of nucleation were used in the calibration model.

Concentrations between 0.025 and 0.050 g (g solution)^−1^ were measured at temperatures between 16 and 44 °C. Table S1 (see the Supporting Information) summarizes the range of solution conditions of which IR spectra were measured. Figure S1 shows typical FTIR spectra measured for a hexamine–ethanol solution. Two distinct peaks characteristic of hexamine were selected to calibrate the spectra against concentration. These were located between wavenumbers of approximately 1227–1246 and 993–1024 cm^–1^.

Of the total 320 measured spectra, 50 were excluded from the calibration model to use for model validation. A second derivative transformation using a Savitzky–Golay filter with 17 points of smoothing was applied to the spectra before calibration. The PLS model calculated after pre-processing the calibration spectra was applied to the 50 validation spectra. The model-predicted concentrations and actual values are compared in Figure S2, where the data fit an *R*^2^ correlation coefficient of 0.9828.

### 2.4 Characterization of MSZW with Varying Scale

The MSZW was first measured in a 500 mL crystallization vessel. Following the polythermal method,^40^ the vessel temperature was cycled 3 times between 5 and 50 °C at a heating rate of 0.15 °C min^–1^ and cooling rate of −0.3 °C min^–1^. Dissolution and crystallization points were recorded where the turbidity probe measured 100 and <90% transmittance, respectively. The solution concentration was varied by dissolving enough hexamine into 400 g of ethanol to reach the solubility limit at 15, 25, 30, and 40 °C, as calculated using the solubility expression presented in Table 1.

MSZW data was also obtained using a Technobis Crystal16 system, which was fitted with sixteen 1.5 mL vials each with a 7 mm magnetic stirrer. The same temperature ramp procedure as previously described was applied to vials containing around 1.5 mL (1.18 g) of ethanol, with appropriate amounts of hexamine to reach similar saturation points as in the 500 mL vessel. Tuning of the turbidity measurements (automatic adjustment of the brightness) was performed after the first heating step in all cases. Agitation using the magnetic stirrer speeds was set to 900 rpm throughout the full duration.

### 2.5 Process Model Parameterization

The mixed-suspension, mixed-product removal (MSMPR) crystallizer module in gPROMS FormulatedProducts v2.2.0 solves a 1D PBM through discretization of the volume-based CSD by the method of classes.^41^ The CSD is divided into a number of size ranges known as classes with a width of Δ*C*_*i*_ = *L*_*i*_ – *L*_*i–*1_, where *i* denotes a given size class. The number of particles per unit volume within class *C*_*i*_ at time *t* is determined using the following equation
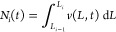
3Integrating this equation and following the same assumptions as before for a batch crystallizer (no flow in or out, growth rate independent of size) results in the following expression that represents the change in particle numbers between classes over time
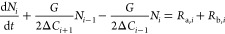
4The flowsheet for a batch cooling crystallization process, built within the software to provide the structure for resolving a 1D PBM for the hexamine–ethanol system, contained a few key features for monitoring the crystallization process. Size distribution and composition sensors were used to closely monitor the CSD and supersaturation, respectively, during the simulation. The types of input data required for each remaining component in this flowsheet are summarized below:

- Global specifications—information about the selected crystallization system, including the components’ molecular weights, physical properties, crystal shape factor, and the solubility curve.
- MSMPR crystallizer—kinetic parameters for the rates of primary nucleation, growth, and secondary nucleation (attrition), equipment size with impeller diameter, speed and power/pumping numbers, and initial composition and mass of liquid.
- Temperature controller—a piecewise linear temperature profile constructed from multiple segments, specifying the start/end temperatures, and durations of each segment.

The solubility and kinetic information listed in Table 1 were implemented into the model, using the nucleation rate expression as attrition-based secondary nucleation and selecting values for the rate constant/supersaturation order in the middle of the reported variance. A logarithmic grid with a resolution of 200 grid points was used to calculate the CSD, with the bounds of crystal size set to 1–1500 μm.

### 2.6 Seeded Linear Cooling Crystallization for Model Validation

A series of seeded linear cooling crystallizations were carried out in the Radleys 500 mL crystallization vessel to investigate the effects of various seeding conditions on the hexamine–ethanol crystallization system. In addition, the resulting concentration and size measurements were used to validate the process model’s CSD prediction.

Two sets of seed crystals were prepared through different methods. 100 g of the raw hexamine from the supplier was sieved by hand through a 250 μm sieve, producing around 50 g of seeds with a volume mean size of 178.4 μm. A second batch of seeds was prepared through milling; 50 g of raw hexamine was put through a ball mill with 1 cm^–3^ yttrium stabilized zirconia milling media for 20 h. This method produced around 40 g of seeds with a volume mean size of 69.4 μm.

At the beginning of each run, hexamine was added to 400 g of ethanol to make up an initial concentration of 0.0507 g (g solution)^−1^ (saturation temperature of around 42 °C). The solution was heated to 50 °C and held for 60 min to ensure all material had dissolved. The solution was then rapidly cooled to the selected seeding temperature, and dry solid seed crystals were added. A constant temperature was held for 30 min to allow the system to equilibrate, before cooling at the selected linear cooling rate down to 7 °C.

Five crystallization experiments were performed in total, the conditions of which are summarized in Table 2. These were selected such that comparisons could be made between specific conditions through different pairs of experiments, including cooling rate (Runs A and D), seed type/size (Runs A and B), seeding temperature (Runs B and E), and seed mass (Runs B and C). The solution concentration was measured in situ using the ATR-FTIR probe. Additionally, the particle count was monitored during Runs A, B, and C using the FBRM probe. Product crystals were removed and filtered under vacuum using a Buchner funnel and their shape and size were analyzed using a Malvern Morphologi G3 and an Olympus BX51 fluorescence microscope.

**Table 2:** Process Conditions Applied to Each Seeded Linear Cooling Crystallization Run

	Run A	Run B	Run C	Run D	Run E
seeding temperature (°C)	40	40	40	40	38
cooling rate (°C min^–1^)	0.2	0.2	0.2	0.3	0.2
seed type	sieved	milled	milled	sieved	milled
seed mean size (μm)	178.4	69.4	69.4	178.4	69.4
seed mass (g)	1	1	3	1	1

Runs D and E were selected as cases for model validation. To simulate these experiments using the gPROMS process model, the measured temperature profiles were reconstructed in the temperature controller of the model flowsheet. The initial seed CSDs were input through two methods: the measured volume CSD from the Morphologi G3, or a lognormal peak that most closely represented the measured CSD. The *D*_50_ and standard deviation of this peak for the sieved seeds were 199 and 40 μm, respectively, whereas for the milled seeds they were 58 and 30 μm, respectively. Simulations of these batches were run with a time interval of 5 s. The full range of input data for the Global Specifications, MSMPR Crystallizer and Temperature Controller components of the gPROMS flowsheet are summarized in Tables S2–S4 (see the Supporting Information).

### 2.7 Growth Rate Parameter Estimation

After evaluating the performance of the process model using the growth kinetics obtained from the literature, the parameter estimation feature in gPROMS software was used to investigate whether a better fit to experimental data could be achieved. In addition to reestimating the parameters for the power law growth expression, implementation of the two-step growth expression was also tested.

The reestimated growth rate parameters were found to be unreliable as to whether model predictions were improved or worsened depending on the operating conditions. Therefore, the reestimated parameters were rejected and the original model parameters given in Table 1 were retained. The full procedure and results of the parameter estimation exercise can be found in Section 1 in the Supporting Information.

### 2.8 Supersaturation MPC Development Using PharmaMV

**Figure 2:**
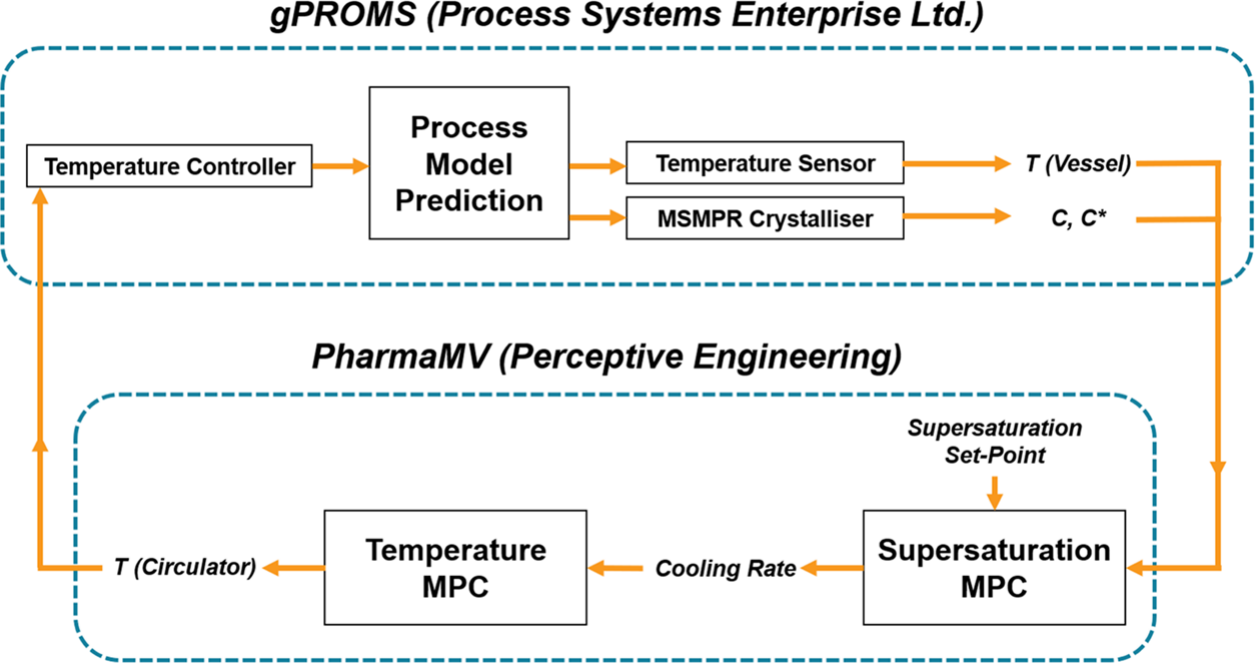
Data flow within the gPROMS/PharmaMV digital twin during a supersaturation control simulation.

Most information in the PharmaMV real-time control software is treated as either an actuation signal (sent from PharmaMV to external software/equipment) or a measured signal (calculated by PharmaMV or measured from external source). A typical MPC “block” within PharmaMV will involve a statistical relationship being calculated between an actuation signal and one or more measured signals. The MPC strategy for supersaturation control used two of these blocks that had been built as part of the CrystalMV template by Perceptive Engineering Ltd. The gPROMS process model was connected with PharmaMV to provide the necessary temperature, concentration, and other process measurement data, as described by the diagram in Figure 2.

A batch crystallization simulation in gPROMS and data sampling in PharmaMV were run simultaneously, where the latter received signals for the current temperature and supersaturation within the vessel. The first MPC block compared the current supersaturation with a manual set-point value, manipulating the cooling rate as a control move to drive the necessary change in supersaturation. This cooling rate informed the second MPC block to make an appropriate control move on the circulator temperature, in turn affecting the vessel temperature in relation to a PI controller simulated within gPROMS. Data was transferred within the digital twin at a time interval of 5 s, stopping automatically when a set end temperature was reached. The temperature and supersaturation MPC blocks made decisions to update their associated manipulated variables every 15 and 60 s, respectively.

Each MPC block was trained through pseudo-random binary sequence (PRBS) testing, where the corresponding manipulated variable for each controller was varied between set limits during a gPROMS process simulation. The resulting changes in the controlled variables were statistically calibrated to an autoregressive with exogenous input (ARX) model structure using the recursive least squares (RLS) method.^42^ For the temperature MPC, the circulator temperature was held constant and stepped between 5 and 50 °C. For the supersaturation controller, a cooling ramp was run starting from 40 °C to generate supersaturation and the cooling rate was stepped between 0.1 and 1.5 °C min^–1^.

To test the fully trained digital twin, examples of in silico supersaturation control were simulated. Experimental conditions for these simulations such as the supersaturation set-points were chosen based on the results of the seeded linear cooling experiments, which have been listed in the corresponding Section 3.

### 2.9 Validation of Supersaturation MPC Strategy

The MPC strategy for supersaturation control developed in PharmaMV was implemented into a similar crystallization setup as that described in Section 2.2, which was adjusted to allow for automated operation of the circulator through communication with the software platform. Experiments were performed in a Radleys 500 mL jacketed crystallizer with a 40 mm diameter 4-blade pitched blade impeller running at 450 rpm, which matched the conditions used during pervious seeded crystallization experiments. A LAUDA Proline RP 855 refrigerating circulator was used to control the jacket temperature, using water as the coolant. Vessel and circulator temperature signals alongside IR spectra measured using a Mettler Toledo ReactIR spectrometer were connected to a PC dedicated to running the MPC strategy in PharmaMV.

**Figure 3:**
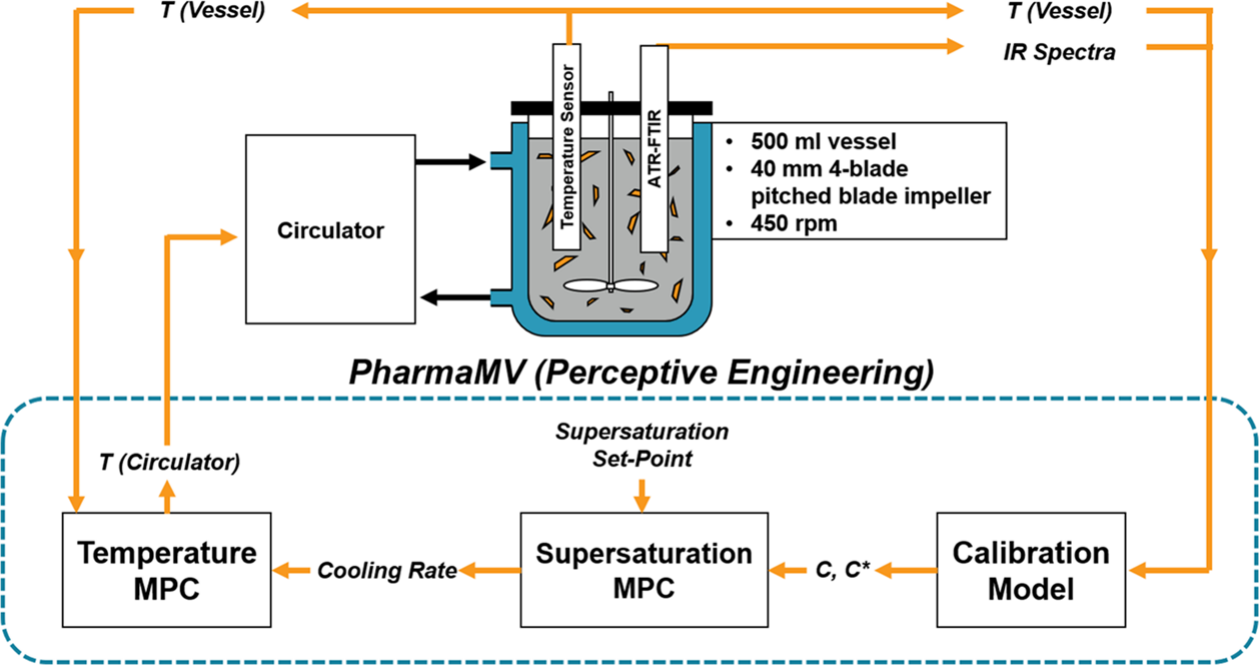
Data flow within the MPC strategy during a physical batch cooling crystallization with supersaturation control.

Figure 3 illustrates how the MPC data flow schematic in Figure 2 was adapted for the physical crystallization setup. A concentration calibration model was developed within PharmaMV software using new spectral measurements with the ReactIR system, following the same procedure and experimental conditions described in Section 2.3. A second derivative transformation using a Savitzky–Golay filter with 10 points of smoothing was applied to the spectra before calibration. The calibration model was incorporated into the overall strategy as its own MPC block, which communicated the measured concentration to the supersaturation MPC block.

The seed crystals used in supersaturation control experiments were produced via wet milling using an IKA magic LAB. The raw hexamine from the supplier was suspended in 500 mL of saturated ethanol solution and pumped continuously through the mill and attached hopper. Using a pipette, 10 mL samples were removed every 5 min to check the progress of milling using a Malvern Mastersizer 3000, stopping the milling process once the desired size had been achieved. Two sets of seed crystals were prepared using different conditions. Using a fine-toothed rotor at a speed of 20 000 rpm for 30 min, approximately 30 g of seeds with a volume mean size of 69 μm were produced. Using a coarse-toothed rotor at 8000 rpm for 20 mins produced approximately 50 g of seeds with a mean size of 112 μm.

## 3 Results and Discussion

### 3.1 MSZW of Hexamine–Ethanol Crystallization System

At both the 1.5 and 500 mL scales, high fluctuations were experienced in the transmittance (the turbidimetric probe output signal) during the initial heating step, improving significantly during dissolution of the recrystallized material in the second temperature cycle. It was thought that this was due to caking of the raw hexamine feed and therefore presence of clumps of material in the initial cycle that caused disruptive readings when passing the turbidity probe, as opposed to the much finer recrystallized material during the second and third cycles due to the abundance of nuclei formed.

The MSZW curves for each scale of crystallizer are plotted in Figure S3 (see the Supporting Information). Using the Crystal16 resulted in an MSZW between 8 and 15 °C, whereas in the 500 mL vessel, a narrower 5–10 °C range was observed. Both appeared to approach the solubility curve at higher temperatures. It was noted that the narrow MSZW at −0.3 °C min^–1^ cooling rate may make seeding within these conditions difficult if primary nucleation was to be avoided; however, the results of the seeded linear cooling batches later disproved this concern.

Dissolution point measurements were generally a good match with the solubility data from the literature. The data point at 15 °C saturation (0.0267 g (g solution)^−1^) showed the largest discrepancy, being measured at 12.9 °C. This was put down to undissolved hexamine trapped within a small vortex below the impeller, undetected by the turbidity probe and therefore indicating an earlier clear point.

### 3.2 Seeded Linear Cooling Crystallizations

**Figure 4:**
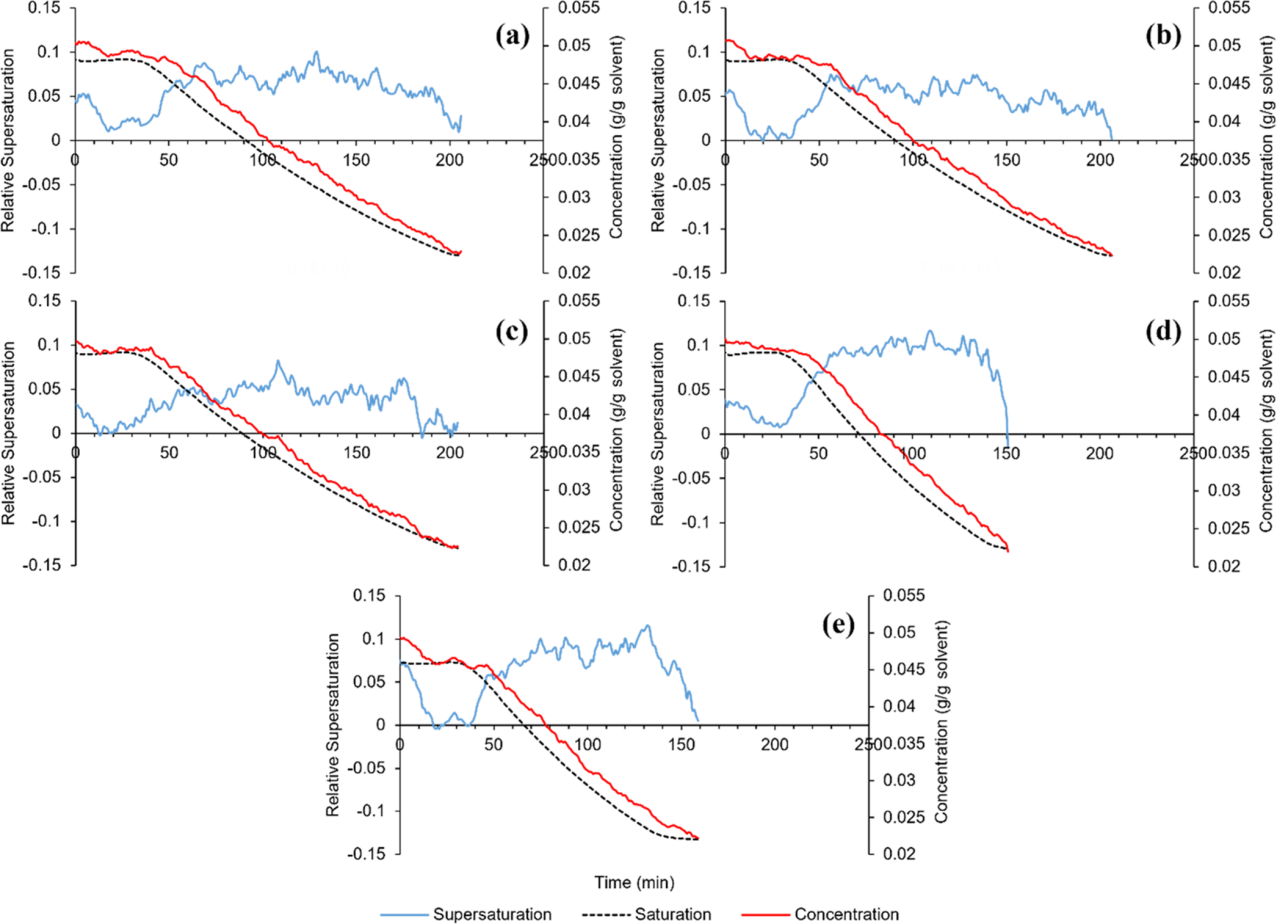
Measured concentration and supersaturation profiles during the seeded linear cooling crystallizations, starting from the point of seeding. (a) Run A, (b) Run B, (c) Run C, (d) Run D, and (e) Run E.

**Figure 5:**
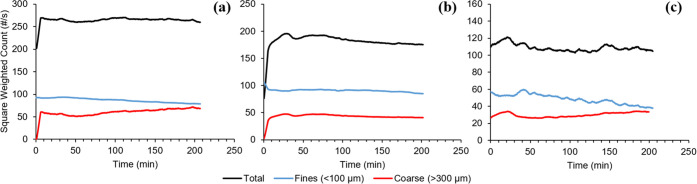
Measured square-weighted particle counts during the seeded linear cooling crystallizations, starting from the point of seeding. (a) Run A, (b) Run B, and (c) Run C.

**Figure 6:**
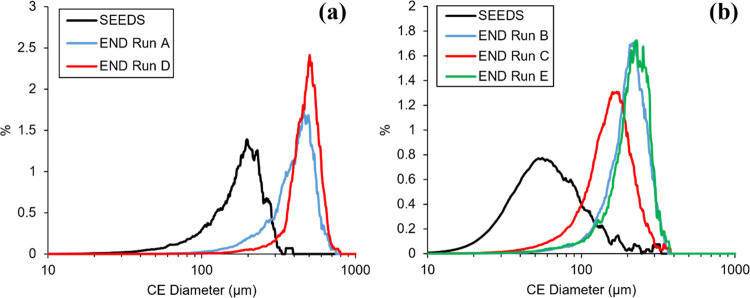
Measured seed and product volume CSD for batch crystallizations using (a) sieved seeds 178 μm volume mean size and (b) milled seeds, 69 μm volume mean size.

The concentration profiles measured by the ATR-FTIR probe during each of the seeded linear cooling crystallizations are presented in Figure 4, alongside the relative supersaturation ((*C* – C*)/C*) that was calculated using the saturation concentration. To improve upon the instability of the concentration measurements, the profiles shown in Figure 4 have been averaged over 5 data points (measured over 5 min). For the three batches that included the FBRM probe, the square-weighted particle counts of fine (<100 μm) and coarse (>300 μm) material are shown in Figure 5. The volume-based CSDs and mean sizes of the product crystals from each batch are shown in Figure 6 and Table 3, respectively.

**Table 3:** Volume Mean Size of Product Crystals from Each Seeded Linear Cooling Crystallization Run

	Run A	Run B	Run C	Run D	Run E
product volume mean size (μm)	407.7	207.4	157.8	478.5	214.4

While most cases do not show a noticeable drop in concentration during the initial holding period after adding the seed crystals, this is much more significant in Run E where a lower seeding temperature was chosen. The resulting mean crystal size was higher than that from Run B, for which all other conditions were identical. This suggested a high importance of the growth rate during the holding period, which would have been faster due to the higher supersaturation generated from choosing a lower seeding temperature.

The saturation point was reached most rapidly in Run C, where a larger mass of seed crystals was added than in Run B. Given the larger surface area available for crystal growth, the supersaturation remained low throughout the entire batch. At a higher seed loading, the reduced final mean crystal size was likely due to the same mass of solute being distributed around a larger number of particles. This is an important consideration for supersaturation control, as a larger seed mass would likely increase difficulty in being able to maintain the supersaturation if the growth rate was too high.

Another key factor that would affect process control is the cooling rate. Comparing Runs A and D, which only differed in cooling rate, it was observed that a lower maximum supersaturation was generated when cooling more slowly. This implied that the potential cooling power achievable through control of the jacket temperature may limit the supersaturation set-points that would be feasible.

From the particle count measured during Runs A, B, and C, it was found that each batch followed the same general trends. The total particle counts remained relatively constant over time, whereas there was a more obvious increase and decrease in the coarse and fine material, respectively. This would suggest that the crystallization of hexamine from ethanol under these conditions was dominated by crystal growth, without any significant secondary nucleation processes occurring.

**Figure 7:**
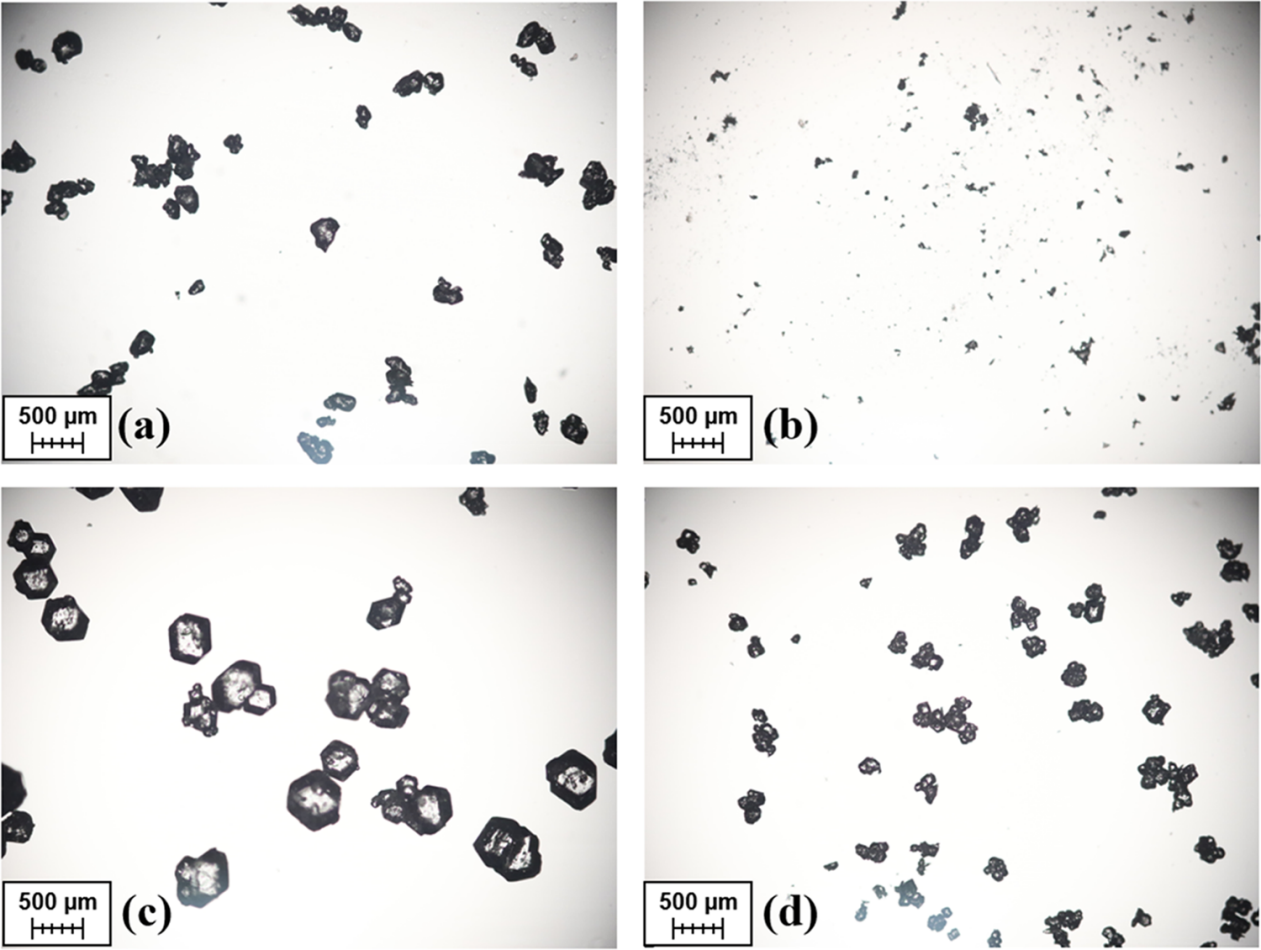
Microscope images of (a) sieved seeds used in Run D, (b) milled seeds used in Run E, (c) product crystals from Run D, and (d) product crystals from Run E.

Microscope images that were taken of the seed and product crystals, such as those from Runs D and E that are shown in Figure 7, further supported the “growth only” hypothesis. There was no obvious increase in the number of fine particles at the end of batches that used both the larger sieved and smaller milled seed crystals.

A more significant difference observed was the increase in agglomeration present when starting with the smaller seeds. Given that no agglomeration in the absence of any kinetic parameters to describe this mechanism was considered in the PBM, experiments involving larger seed crystals would likely have their crystallization behavior more accurately predicted. It would therefore be more favorable to use larger seeds during process control experiments, to ensure the MPC strategy performed well during the initial validation phase.

### 3.3 Crystallization Process Model Validation

**Figure 8:**
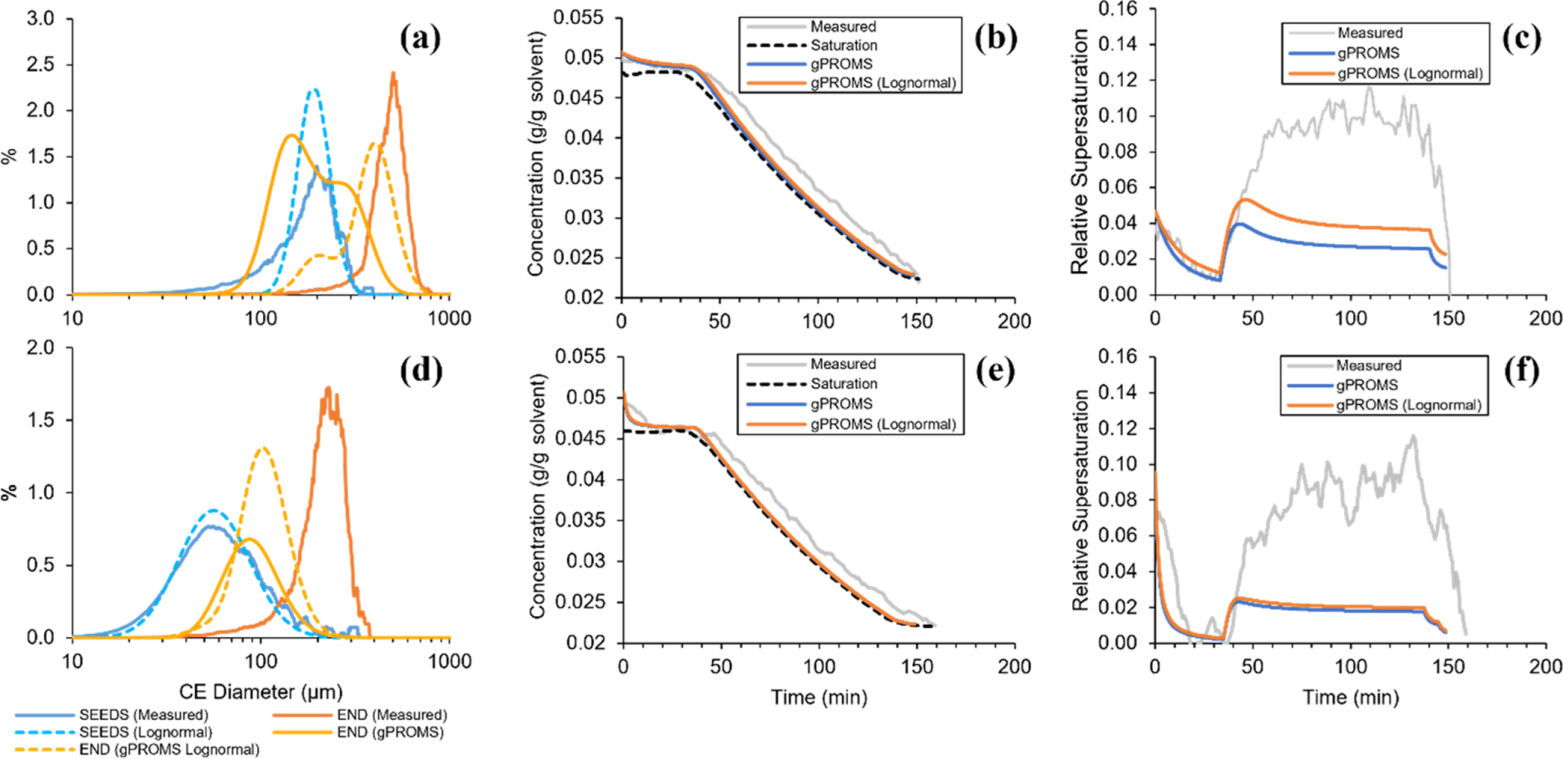
Comparison of CSDs, concentration, and supersaturation profiles predicted by gPROMS with measured data. Simulations starting with either the measured seed CSD or a representative lognormal peak. Results correspond to seeded linear cooling (a–c) Run D and (d–f) Run E.

Batch crystallization Runs D and E were simulated using the kinetic parameters given in Table 1. These batches were selected due to being the most different from each other (seed size, seeding temperature, and cooling rate) to ensure the model was accurate over a wide range of conditions. Figure 8 shows a comparison between the predicted and measured CSDs, concentration profiles, and supersaturation profiles for these batches. When the seed CSD was defined using the measured distribution, the gPROMS process model underpredicted the final crystal size in both cases. Improvements were observed when specifying the seeds as a lognormal peak, which had a more significant effect on Run D due to the elimination of the fine material tail in the distribution. The predicted volume mean crystal size increased from 216 to 393 μm for Run D, and 99 to 113 μm for Run E.

Additionally, an improved agreement with the measured concentration profile during the batch was achieved using the lognormal CSD, most significantly for Run D. Despite this, both cases showed a significant underprediction of the level of supersaturation experienced during the linear cooling section. The impacts of this on the performance of the controllers trained using the digital twin in its current state have been discussed when considering the differences between the in silico and physical supersaturation control.

### 3.4 In Silico MPC of Supersaturation

To test the fully trained digital twin, examples of in silico supersaturation control were simulated. Table 4 summarizes the process conditions applied in these simulations. The parameters describing the seed CSD were selected based on those produced for physical validation, as described in the following section. During each simulation, the temperature was held at the initial value for 30 min before switching on both of the MPC blocks in Figure 2.

**Table 4:** Process Conditions Applied to the gPROMS/PharmaMV Digital Twin for Supersaturation MPC Performance Testing

crystallizer volume (mL)	500
*D*_50_ of seed CSD (μm)	110
standard deviation of seed CSD (μm)	25
initial seed mass (g)	1
initial temperature (°C)	40
end temperature (°C)	5
supersaturation set-point	0.012, 0.024, 0.036

**Figure 9:**
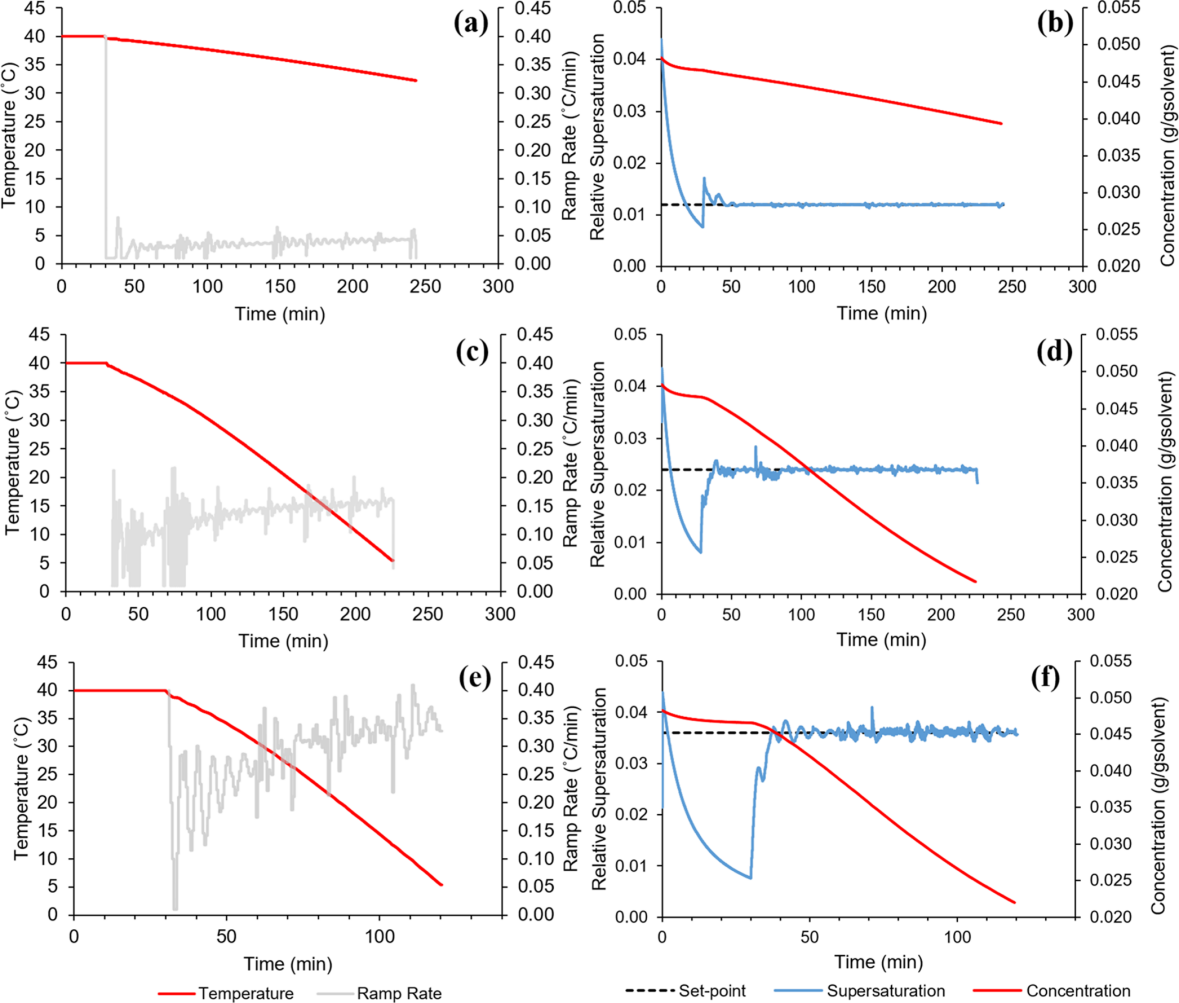
Temperature, cooling (ramp) rate, concentration, and supersaturation profiles generated during in silico supersaturation MPC, for supersaturation set-points of (a, b) 0.012, (c, d) 0.024, and (e, f) 0.036.

The cooling and supersaturation profiles resulting from the in silico supersaturation control simulations at three different supersaturation set-points are presented in Figure 9. The set-points were chosen based on the maximum values reached during the seeded linear cooling experiments. In each case, the supersaturation decreased as expected during the initial 30 min holding period, where the seeds crystals were left to grow. The increase in supersaturation at the beginning of cooling would typically result in an offshoot above the set-point, which the MPC strategy was able to correct by manipulating the cooling rate. The circulator set-point temperature and measured circulator and vessel temperatures always overlapped; therefore, only a single temperature profile has been plotted in Figure 9. This is due to the gPROMS process model assuming almost instantaneous heat transfer dynamics (i.e., no time delay between change in circulator and response in vessel temperatures), which were captured when training the temperature MPC block.

For the lowest set-point of 0.012, the simulation was stopped early due to the anticipated length of the batch time. Although the level of control was very good after the initial offshoot, it was expected based on the cooling rates chosen by the controller that the conditions would lead to a long batch that may be considered ineffective from an industrial viewpoint. Experiments were completed in a more reasonable time for the remaining set-points, due to the overall higher cooling rates required to maintain higher supersaturations. There was a noticeable increase in the instability of control as the set-point increased, caused by the more aggressive changes made to the cooling rate by the supersaturation MPC block.

The tuning weights within the MPC block could be adjusted to reduce these unstable fluctuations in supersaturation by preventing large changes in the cooling rate. However, a variable tuning would have to be applied to dampen these responses only when the measured supersaturation is close to the set-point. Otherwise, the MPC strategy would take too long to bring the supersaturation up to the set-point in the early stages of the crystallization process.

### 3.5 Physical MPC of Supersaturation for Validation

At the beginning of each physical control run, hexamine was added to 400 g of ethanol to make up an initial concentration of 0.0507 g (g solution)^−1^. The solution was heated to 50 °C and held for 60 min to ensure all material had dissolved. The solution was cooled at the maximum rate achievable by the circulator (−0.6 °C min^–1^) down to 40 °C, where 1 g of the 112 μm dry seed crystals were added. Following this, the crystallizer was held at a constant temperature for 30 min to allow the system to equilibrate, after which the temperature and supersaturation MPC blocks in PharmaMV were switched on.

Starting with an initial cooling rate of −0.4 °C min^–1^, the MPC strategy compared the predicted supersaturation trajectory to in situ measurements via the ReactIR to determine the cooling rate required to maintain the supersaturation close to the selected set-point. The cooling rate was updated by the control block every 30 s, continuing until a final temperature of 5 °C was reached. Each of the three supersaturation set-points (0.012, 0.024, 0.036) chosen for the in silico simulations was applied to the physical setup for validation.

**Figure 10:**
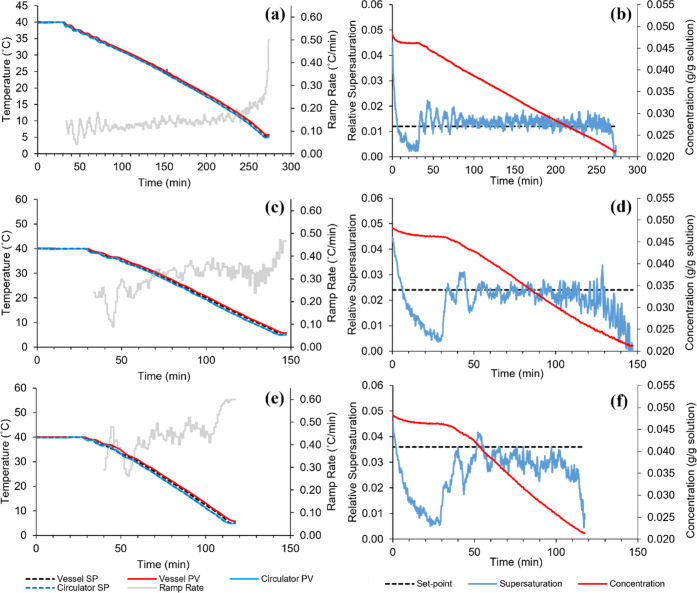
Temperature, cooling rate, concentration, and supersaturation profiles generated during physical supersaturation MPC, for supersaturation set-points of (a, b) 0.012, (c, d) 0.024, and (e, f) 0.036.

The cooling and supersaturation profiles resulting from supersaturation control experiments performed on the 500 mL jacketed crystallizer at CMAC are presented in Figure 10. To assess the performance of the MPC strategy, the means and standard deviations of the measured supersaturation profiles have been calculated and listed in Table 5, alongside those calculated from the in silico simulated profiles for the same conditions.

**Table 5:** Mean and Standard Deviation of Supersaturation during In Silico Simulations and Physical Validation Experiments for Supersaturation Control

	supersaturation mean		supersaturation std. dev.	
supersaturation set-point	in silico	experimental	in silico	experimental
0.012	0.0121	0.0137	4.79 × 10^–4^	2.03 × 10^–3^
0.024	0.0239	0.0227	4.76 × 10^–4^	3.15 × 10^–3^
0.036	0.0359	0.0300	8.05 × 10^–4^	4.00 × 10^–3^

Generally, it was observed that higher cooling rates were required to reach and maintain a given supersaturation set-point compared with simulations. At a supersaturation set-point of 0.012, an approximately doubled cooling rate meant that the final temperature of 5 °C was reached in a much shorter time than expected. Higher cooling rates often led to more unstable control, given the work required by the circulator to respond to its constantly changing temperature set-point. This may have been a contributing factor toward the noticeable increase in instability of the supersaturation from simulations to physical experiments, as reflected by the standard deviations being an order of magnitude higher. The trend of increasing instability at higher supersaturation set-points matched that observed in simulations.

Considering potential factors related to the software, the heat transfer dynamics captured by the training data provided to each MPC block may have been a cause of instability. The temperature MPC block could be adjusted to match the dynamics achievable by the circulator and jacketed vessel quite easily. However, the gPROMS model-trained supersaturation MPC block was still operating under the assumption of perfect temperature control. This meant that the MPC strategy would expect an immediate change in supersaturation upon choosing a new cooling rate, which was not feasible to achieve in the physical experimental setup. The mean supersaturation values were much further from the set-points for the physical experiments, as expected when considering the disturbances caused by imperfect temperature control and concentration measurement accuracy and precision. With a supersaturation set-point of 0.012, this was the only case where the mean value exceeded the set-point. With the concentration declining very gradually due to slower growth rates at low supersaturations, it is likely that the system was more sensitive to increases in supersaturation when the cooling rate was set too high.

The opposite effect could explain why the mean supersaturation fell short of the higher set-points of 0.024 and 0.036; in these cases, the system appeared to be more sensitive to a drop in supersaturation should the cooling rate be insufficient. For the highest set-point, the MPC strategy struggled to bring the supersaturation up to this value for most of the batch. This suggested that a supersaturation of 0.036 may have been above the limit achievable by the cooling rates allowed by the MPC strategy. These cooling limits could not be increased without creating a large temperature differential between the vessel and circulator temperatures, which would have made precise control over the cooling rate difficult to maintain.

## 4 Conclusions

The hexamine–ethanol crystallization system was characterized through a series of unseeded and seeded cooling crystallizations performed at various scales. The MSZW measured at −0.3 °C min^–1^ cooling rate was narrower in a 500 mL jacketed crystallizer compared with the 1.5 mL Crystal16 system. Despite this, the operating conditions chosen for the seeded crystallization experiments were typically able to avoid both primary and secondary nucleation from occurring. It was found that seed crystals with a mean size greater than 100 μm were most suitable for investigating the performance of process control, as these would typically grow into the expected morphology without significant agglomeration. A low mass was also preferable to prevent an overly rapid decline in supersaturation when attempting to control this parameter.

The nucleation and growth rate kinetics used to parameterize the 1D PBM in gPROMS FormulatedProducts proved to give a reasonable prediction of the change in solution concentration during a seeded linear cooling crystallization. The crystal size was often underpredicted; however, this was improved slightly through the use of lognormal CSDs for the initial seed crystals. It is hypothesized that the introduction of agglomeration kinetics into the gPROMS model would improve its predictions of the crystal size. However, no expression to describe hexamine’s unique agglomeration mechanism currently exists, and the quantification of such an expression was outside the scope of this study.

The gPROMS process model was used to train two RLS model predictive controllers in PharmaMV. The temperature MPC block managed the relationship between the vessel and circulator temperatures, whereas the supersaturation MPC block managed the relationship between supersaturation and cooling rate. The digital twin formed by pairing both software allowed for in silico simulations of supersaturation control over a virtual 500 mL jacketed vessel. For supersaturation set-points between 0.012 and 0.036, the overall performance of the controllers was excellent. It was noted that stability decreased slightly with increasing set-point values; however, this could easily be corrected through tuning of the supersaturation MPC block to prevent large moves in the cooling rate when the set-point error is low. These results have proven that, despite some imperfections in the PBM, the MPC strategy was capable of adapting to the simulated measured data and theoretically control supersaturation at varying levels.

When the MPC strategy was implemented on a physical 500 mL crystallizer, it was found that higher cooling rates were required to reach the same supersaturation set-points as the in silico simulations achieved. The highest set-point of 0.036 was found to be too high for the MPC strategy to maintain, given the limits of the circulator’s cooling power. Fortunately, the remaining two supersaturation targets were achieved at a reasonably good level of performance, based on the mean and standard deviation values calculated from the measured supersaturation profiles. The stability of the control was expectedly lower due to disturbances resulting from imperfect heat transfer dynamics and the precision of concentration measurements.

Overall, the supersaturation control capability of the MPC strategy is promising for a single-component crystallization system. Should the accuracy of control be equally sufficient when following a variable supersaturation set-point (i.e., the optimal supersaturation profile to achieve a target crystal size), the strategy would be a valuable tool for industrial crystallization processes. A particularly beneficial feature is the adaptability of the digital twin, which could easily be adjusted for a more complex solute–solvent system than the model system chosen for this study.

## Supporting Information Available

Calibration of FTIR spectra to hexamine–ethanol solution concentration; MSZW at 0.3 °C/min; input data for the gPROMS process model; and reestimation of the growth rate parameters (PDF)

## Nomenclature

*B*: birth rate (breakage) (#(s·m^3^)^−1^)
*C*: concentration (g (g solution)^−1^)
*C**: saturation concentration (g (g solution)^−1^)
Δ*C*_*i*_: width of size class *i* (m)
*D*: death rate (agglomeration) (#(s·m^3^)^−1^)
*D*_AB_: diffusion coefficient (m^2^ s^–1^)
*d*_m_: molecular diameter of solute (m)
*G*: growth rate (m s^–1^)
*g*: growth rate supersaturation order
*J*: nucleation rate (#(s·m^3^ solvent)^−1^)
*K*_v_: volume shape factor of crystals
*k*: Boltzmann constant (J K^–1^)
*k*_d_: mass transfer coefficient (m s^–1^)
*k*_g_: growth rate constant (m s^–1^)
*L*: characteristic length (crystal size) (m)
*N_i_*: number of particles in size class I (#m^−3^)
*n*(*L*): number density function (#(m·m^3^ slurry)^−1^)
*R*_a,*i*_: net agglomeration rate in size class I (#(s·m^3^)^−1^)
*R*_b,*i*_: net breakage rate in size class I (#(s·m^3^)^−1^)
*T*: temperature (°C or K)
*t*: time (s)
*v*(*L*): volume density function (m^3^ crystal (m·m^3^ slurry)^–1^)
α: diffusivity correction factor
ε: energy dissipation rate (m^2^ s^–3^)
η: dynamic viscosity (kg (m·s)^−1^)
υ: kinematic viscosity (m^2^ s^–1^)

## Abbreviations

1D: one-dimensional
ARX: autoregressive with exogenous input
ATR: attenuated total reflection
BFDH: Bravais–Friedel–Donnay–Harker
CFD: computational fluid dynamics
CSD: crystal size distribution
FBRM: focused-beam reflectance measurement
FTIR: Fourier transform infared
MPC: model predictive control
PBM: population balance model
PI: proportional-integral
PLS: partial least squares
RLS: recursive least squares
