# Supplementary material for: Development of a Digital Twin for the Prediction and Control of Supersaturation during Batch Cooling Crystallization
Source: Ind Eng Chem Res. 2023 Jul 3;62(28):11067–81. doi: 10.1021/acs.iecr.3c00371 (PMC10360059; doi:10.1021/acs.iecr.3c00371)
Supplement: Supplementary file 1 — ie3c00371_si_001.pdf [file ie3c00371_si_001.pdf]

Development of a Digital Twin for The Prediction and Control of Supersaturation During Batch Cooling Crystallisation

Ryan Leeming[1], Tariq Mahmud[1*], Kevin J. Roberts[1], Neil George[2], Jennifer Webb[2], Elena Simone[3], Cameron J. Brown[4]

1. School of Chemical and Process Engineering, University of Leeds, Leeds, LS2 9JT, UK

2. Syngenta, Jealott's Hill, Bracknell, RG42 6EY, UK

3. Department of Applied Science and Technology, Politecnico di Torino, Torino, 10129, Italy

4. CMAC Future Manufacturing Research Hub, University of Strathclyde, Glasgow, G1 1RD, UK

**Corresponding author* (t.mahmud@leeds.ac.uk)

Table S1. Solution Temperatures and Concentrations for which IR Spectra were Measured for the Calibration Model in HorizonMB Software.

Temperature (°C)	Concentration (g/g solvent)						
	0.050	0.045	0.040	0.037	0.034	0.030	0.025
44	X						
42	X						
40	X	X					
38	X	X					
36		X	X				
34		X	X				
32			X	X	X		
30			X	X	X		
28				X	X		
26				X	X	X	
24				X	X	X	
22					X	X	
20						X	X
18						X	X
16						X	X

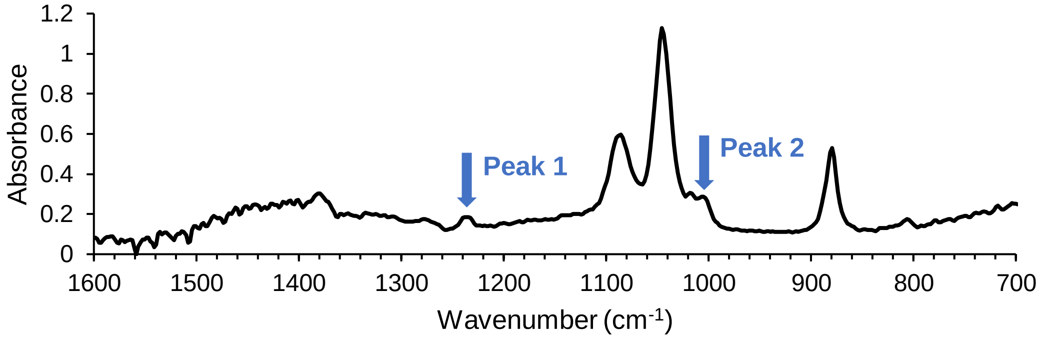

Figure S1. Hexamine-ethanol FTIR spectra measured using the ABB MB3000 spectrometer at 36 °C and 0.04 g/g solvent. Highlighted peaks are those characteristic of hexamine selected for the calibration model.

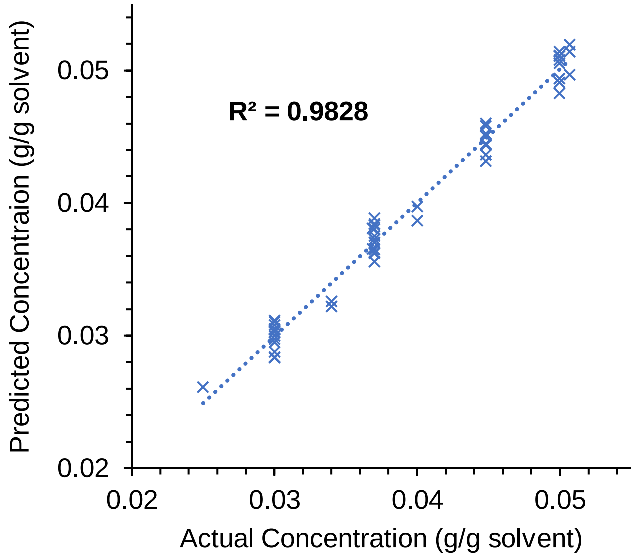

Figure S2. Actual vs. predicted concentrations for the PLS calibration model calculated in HorizonMB software.

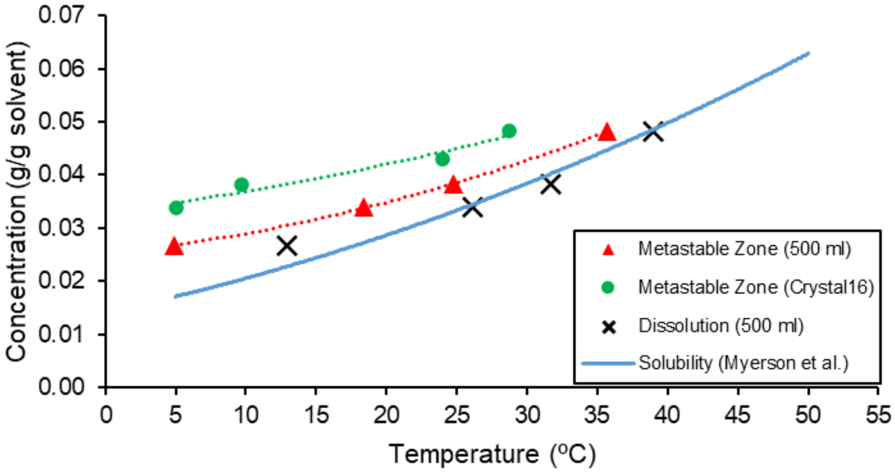

Figure S3. Metastable zone for hexamine-ethanol system obtained from Crystal16 and 500ml vessel using cooling rate of -0.3 ºC/min, solubility from Myerson et al.[1]

Table S2. Input Data for the 'Global Specifications' Component of the Flowsheet for Hexamine-Ethanol Batch Cooling Crystallisation in gPROMS FormulatedProducts.

Section/Property	Input	Source
**System information**		
Components	Hexamine, Ethanol	-
**Liquid properties**		
Molecular weights (kg/mol)	Hexamine: 0.140186<br>Ethanol: 0.04607	-<br>-
Mass density coefficients<br>a, b, c	1172.3, -1.8681, 0.002	Measured
Mass specific heat coefficients<br>Hexamine: a, b<br>Ethanol: a, b, c, d	-402.78, 6.1976<br>6271.4, 93.036, -0.3528, 0.0005	Measured<br>Miyazawa et al.[2]
Dynamic viscosity coefficients<br>a, b, c, d	0.0733, -0.0006, 2×10^{-6}, -2×10^{-9}	Kaye and Laby[3]
**Crystal properties**		
Volumetric shape factor	Equivalent to a sphere	-
Mass density (kg/m^3)	1330	Rohani and Bourne[4]
**Solubility**		
Solubility calculated using	Generic polynomial	-
Solubility coefficients<br>a, b, c	5.354×10^{-1}, -4.13×10^{-3}, 8.2×10^{-6}	Myerson et al.[1]
**Grid parameters**		
Number of grid points	200	-
Grid type	Logarithmic	-
Minimum particle size (µm)	0.1	-
Maximum particle size (µm)	1500	-

Table S3. Input Data for the 'MSMPR Crystalliser' Component of the Flowsheet for Hexamine-Ethanol Batch Cooling Crystallisation in gPROMS FormulatedProducts. Nucleation and Growth Kinetic Parameters are Provided from Myerson et al.[1]

Section/Property	Input				
**Equipment and operation**					
Equipment volume (L)	0.5				
Liquid and solid outflow	Batch operation				
Specific power output	Calculated				
Impeller diameter (m)	0.04				
Impeller frequency (rpm)	450				
Impeller power number	1.3				
Impeller pumping number	0.8				
**Energy balance**					
Heat transfer	Not considered (Adiabatic)				
**Secondary nucleation (attrition)**					
Secondary nucleation (attrition)	Power law kinetics				
Driving force	Absolute supersaturation				
Rate constant (ln(#/m^3.s))	31.543				
Supersaturation order	2.6				
Activation energy (J/mol)	0				
Specific power input order	0				
Minimum particle size (μm)	0				
**Growth and dissolution**					
Growth and dissolution	Power law kinetics				
Driving force	Absolute supersaturation				
Size dependent solubility	Inactive				
Growth rate constant (m/s)	2.6×10^{-2}				
Supersaturation order	1.95				
Activation energy (J/mol)	0				
**Initial conditions**	**Run A**	**Run B**	**Run C**	**Run D**	**Run E**
Mass (kg)					
Solid crystal phase	0.001	0.001	0.003	0.001	0.001
Liquid phase	0.42028	0.42028	0.42028	0.42028	0.42028
Temperature (˚C)	40	40	40	40	38
**Initial conditions: Liquid**					
Mass fraction					
Ethanol	0.95175	0.95175	0.95175	0.95175	0.95175
Hexamine	0.04825	0.04825	0.04825	0.04825	0.04825
**Initial conditions: Solid**					
Specify CSD	As a density distribution function				
Location parameter (μm)	199	58	58	199	58
Standard deviation (μm)	40	30	30	40	30

Table S4. Input Data for the 'Temperature Controller' Component of the Flowsheet for Hexamine-Ethanol Batch Cooling Crystallisation in gPROMS FormulatedProducts.

	Interval 1	Interval 2	Interval 3
**Run A**			
Duration (min)	32	165	2
Final Temperature (˚C)	40	7	7
**Run B**			
Duration (min)	32	165	2
Final Temperature (˚C)	40	7.1	7
**Run C**			
Duration (min)	32	165	2
Final Temperature (˚C)	40	7.1	7
**Run D**			
Duration (min)	33	107.3	8
Final Temperature (˚C)	40	7.8	7
**Run E**			
Duration (min)	35	102.7	7
Final Temperature (˚C)	38	7.2	7

Supplementary Section 1. Re-estimation of Growth Rate Parameters

After evaluating the performance of the process model using the growth rate kinetics obtained from literature, the parameter estimation feature in gPROMS software was used to investigate whether a better fit to experimental data could be achieved.

The crystal growth kinetics reported by Myerson et al.[1] follow the power-law growth expression:

$$G = k_g \Delta C^g \tag{S1}$$

Another expression available in gPROMS is the two-step growth expression[5], which introduces a diffusion-controlled mass transfer step with the following evaluation of the mass transfer coefficient k_d:

$$k_d L = \frac{D_{AB}}{L}\left[\left(\frac{\bar{\varepsilon}L^4}{\upsilon_L^3}\right)^{0.2}\left(\frac{\upsilon_L}{D_{AB}}\right)^{0.33}\right] \tag{S2}$$

$$D_{AB} = \alpha \frac{kT}{6\pi\eta \frac{d_m}{2}} \tag{S3}$$

In total, three different methods of parameter estimation were employed:

- Re-estimation of the rate constant k_g for the power law growth expression, keeping the supersaturation order fixed

- Re-estimation of the supersaturation order g for the power law growth expression, keeping the rate constant fixed
- Estimation of the diffusivity correction factor α for the two-step growth expression, keeping the rate constant and supersaturation order fixed

The parameter estimation method built into gPROMS follows the optimisation of a maximum likelihood function, solved using a non-linear sequential quadratic programming (SQP) algorithm[6]. The objective function can be described as follows[7]:

$$\Phi = \frac{N}{2}\ln(2\pi) + \frac{1}{2}\min_{\theta}\left\{\sum_{i=1}^{NE}\sum_{j=1}^{NV_i}\sum_{k=1}^{NM_{ij}}\left[\ln\left(\sigma_{ijk}^2\right) + \frac{\left(\tilde{z}_{ijk} - z_{ijk}\right)^2}{\sigma_{ijk}^2}\right]\right\} \quad \text{(S4)}$$

This overall function consists of a constant term, variance term and residual term. For a fixed number of experiments and variance model, the constant and variance terms remain fixed during the parameter estimation. Therefore, only the contribution from the residual term is minimised for each of the measured variables included in the optimisation problem:

$$\Phi = \min_{\theta}\left\{\sum_{i=1}^{NE}\sum_{j=1}^{NV_i}\sum_{k=1}^{NM_{ij}}\left[\frac{\left(\tilde{z}_{ijk} - z_{ijk}\right)^2}{\sigma_{ijk}^2}\right]\right\} \quad \text{(S5)}$$

Each parameter estimation was applied using the experimental data from seeded Runs A, B and C. After inputting the temperature profiles for each performed experiment, the software aimed to vary the selected parameters until a good fit was made between the measured concentration profiles and the predicted profiles constructed by the process model. The variance model for concentration in each case was a constant variance of 0.0005 g/g solution, which was chosen based on the precision error of the ATR-FTIR calibration model. As only the initial and final CSDs had been measured, there was insufficient data to also include a size-related property as an additional optimisation objective.

Figure S4 shows a comparison between the concentration predictions for Runs D and E obtained through re-estimation of various growth rate parameters. An evaluation of the percentage error for each parameter estimation method is shown in Figure S5 for both runs. The predicted volume mean sizes of the product crystals for each case are listed in Table S5.

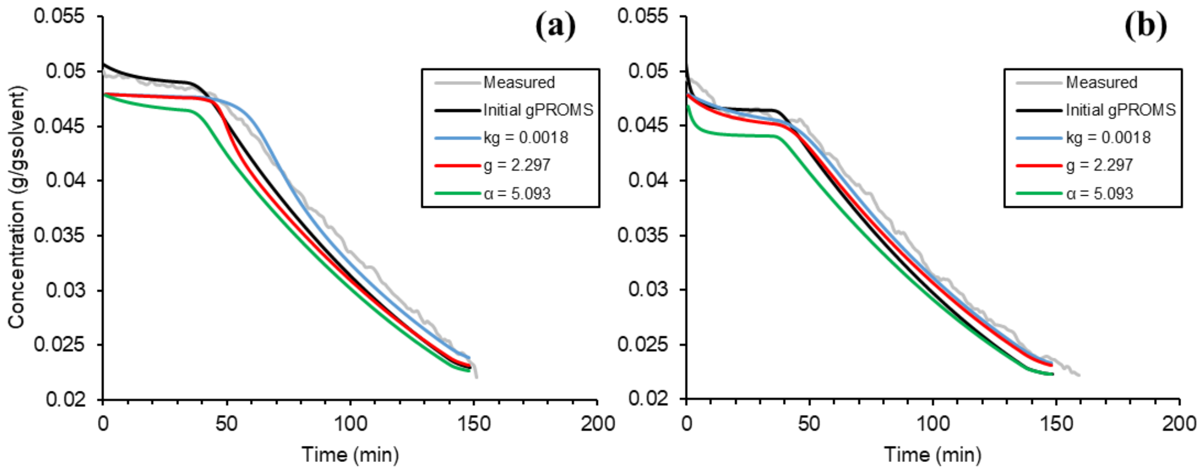

Figure S4. Predicted concentration fit to measured data from a) Run D and b) Run E for gPROMS simulations using re-estimated growth rate parameters.

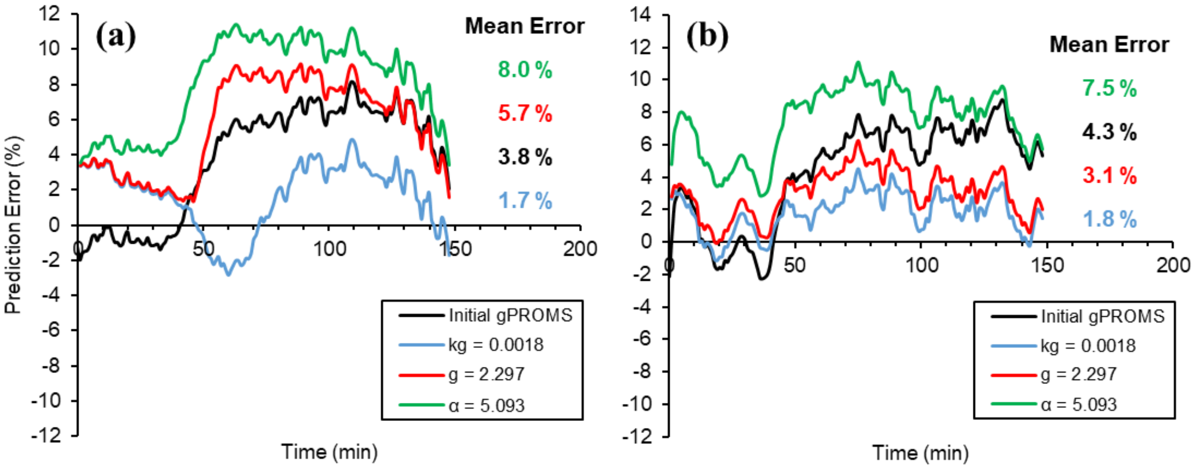

Figure S5. Percentage error in predicted vs measured concentration data from a) Run D and b) Run E for gPROMS simulations using re-estimated growth rate parameters.

Table S5. Predicted Volume Mean Crystal Size of Product from gPROMS Simulations Using Re-estimated Growth rate Parameters.

	**Volume Mean Size (μm)**	
	**Run D**	**Run E**
Measured	478.5	214.4
Initial gPROMS prediction (power-law growth rate, $k_g = 2.60$, $g = 1.95$)	393.0	113.0
Power-law growth rate, estimated $k_g = 0.0018$	181.4	121.7
Power-law growth rate, estimated $g = 2.297$	75.6	121.8
Two-step growth rate, estimated $\alpha = 5.093$	152.7	83.4

For both of the selected validation cases, transitioning from a power law to two-step growth expression proved to weaken the model's prediction of both concentration (3-4 % increase in mean error) and crystal size.

The re-estimated supersaturation order g led to a closer fit to measured data in both concentration (1.2 % decrease in mean error) and size for Run E. However, this was not reflected by the comparison for Run D, where there was a 1.9 % increase in concentration error and significant underprediction of the final mean crystal size, which was around five times smaller that the already underpredicted original value. Despite the re-estimated rate constant k_g leading to an improved concentration fit for both validation cases (1.3-2.1 % decrease in mean error), a similarly poor prediction of the crystal size was observed for Run D.

Given that the crystal size was only measured at the start and end of the batches, the parameter estimation could not fit to this parameter due to the lack of data points. Therefore, a heavy weight was placed on fitting the concentration to the measured profiles that were provided, neglecting the effect on the final crystal size reached. As these results suggested unreliability as to whether the new growth rate parameters would improve the model predictions depending on the operating conditions, the re-estimated parameters were rejected and the original model parameters were retained.

NOMENCLATURE

g	Growth rate supersaturation order	-
k_g	Growth rate constant	m/s
N	Number of measurements taken over all experiments	-
NE	Number of experiments	-
NM_{ij}	Number of measurements of variable j in experiment i	-
NV_i	Number of measured variables in experiment i	-
z_{ijk}	k^{th} predicted value of variable j in experiment i	-
$\check{z}_{ijk}$	k^{th} measured value of variable j in experiment i	-
α	Diffusivity correction factor	-
σ^2_{ijk}	Variance of the kth measurement of variable j in experiment i	-
Φ	Maximum likelihood objective function	-

REFERENCES

(1) Myerson, A. S.; Decker, S. E.; Weiping, F. Solvent selection and batch crystallisation. *Ind. Eng. Chem. Process Des. Dev*. **1986,** *25,* 925-929.

(2) Miyazawa, T.; Kondo, S.; Takuya, S.; Sato, H. Specific heat capacity at constant pressure of ethanol by flow calorimetry. *J. Chem. Eng. Data*. **2012,** *57,* 1700-1707.

(3) Kaye, G. W. C.; Laby, T. H. *Tables of Physical and Chemical Constants*; Longman, 1995.

(4) Rohani, S.; Bourne, J. R. A simplified approach to the operation of a batch crystallizer. *Can. J. Chem. Eng*. **1990,** *68,* 799-806.

(5) Garside, J.; Mersmann, A.; Nyvlt, J. *Measurement of Crystal Growth Rates*; European Federation of Chemical Engineering, Working Party on Crystallization, 1990.

(6) Gill, P. E.; Murray, W.; Saunders, M. A.; Wright, M. H. *User's Guide for NPSOL 5.0: A Fortran Package for Nonlinear Programming*; USA, 2001.

(7) Siemens-PSE Ltd. *gPROMS FormulatedProducts v2.2.0 Documentation.* (Accessed 2023-03-07).
